# Characterization of a Novel Mutation in NS1 Protein of Influenza A Virus Induced by a Chemical Substance for the Attenuation of Pathogenicity

**DOI:** 10.1371/journal.pone.0121205

**Published:** 2015-03-20

**Authors:** Kohei Sasaki, Kyoko Hayashi, Jung-Bum Lee, Fumiya Kurosaki, Toshimitsu Hayashi

**Affiliations:** 1 Graduate School of Medicine and Pharmaceutical Sciences for Research, University of Toyama, Toyama, Toyama, 930–0194, Japan; 2 Research Institute of Life and Health Sciences, Chubu University, Kasugai, Aichi, 487–8501, Japan; University of Hong Kong, HONG KONG

## Abstract

It is generally accepted that live attenuated influenza vaccine (LAIV) has the potential for use as a vaccination against flu. In this study, we demonstrated the nature of an influenza A virus (IAV) mutant induced by treating the IAV with a stable furan derivative, (1*R*,2*R*)-1-(5’-methylfur-3’-yl)propane-1,2,3-triol (MFPT), which had been isolated from *Streptomyces* sp. strain FV60 with the objective of it being an LAIV candidate. The resulting MFPT-resistant (MFPT^r^) IAVs possessed attenuated pathogenicity *in vitro* and *in vivo* when compared with that of the parent virus (H1N1 subtype, NWS strain). Sequencing analysis revealed that a novel mutation, C490U in *ns* gene (P164S in NS1), was detected in all MFPT^r^ virus clones tested. Therefore, NS1 might be a main target of MFPT, and it was suggested that the P164S mutation contributed to the attenuated pathogenicity of the mutants. Although the phosphatidylinositol 3-kinase (PI3K)/Akt signaling pathway is one of the targets of NS1, the MFPT^r^ virus suppressed the phosphorylation of Akt when compared with the wild-type (WT) virus. It was suggested that this might lead to the subsequent inhibition of the cleavage of PARP-1 and caspase-3, which is important for the progression of apoptosis. At the same time, nucleoprotein (NP) was found to be retained in the nuclei in MFPT^r^ virus-infected cells while nuclear export of NP was detected in WT virus-infected cells. In addition, the expression levels of interferon-β transcripts were significantly decreased in MFPT^r^ virus-infected cells. From these results it can be shown that the mutation, NS1^P164S^, might be one of the key residues to control NS1 function concerning the induction of apoptosis. In conclusion, MFPT induced favorable mutation in the *ns* gene for the attenuation of IAV, and therefore might provide the novel methodology for preparing LAIVs.

## Introduction

Influenza continues to be a serious public health issue in the world. Influenza epidemics result in about 3 to 5 million cases of severe illness, and about 250,000 to 500,000 deaths annually [1]. It is caused by infection of the host by influenza viruses which belong to the family *Orthomyxoviridae* and classified into three different types, that is A, B, and C type viruses. Among these viruses, influenza A viruses (IAVs) exhibit the broad host spectrum including mammalians and birds, and it can evolve into highly pathogenic strains [2]. When the evolved viruses increase to pandemic proportions in a population they can quickly spread world-wide, causing significant mortality. For example, in 2009, the outbreak of a new swine IAV (H1N1) occurred to the extent that the World Health Organization (WHO) declared a global pandemic of H1N1 infection. In addition, a highly pathogenic avian influenza virus (A/H5N1) has also become a public health concern due to its ability to cause severe respiratory illness where the mortality rate is approximately 60% in reported human cases [3].

There are two principal clinical approaches for combating influenza: antivirals and vaccines. Among the antivirals, neuraminidase inhibitors are commonly used for treatment against influenza. However, it is well known that drug-resistant viruses have been frequently reported in clinical fields [4,5]. Therefore, the importance of prophylaxis by vaccination has been increasing. Inactivated influenza vaccines are the most popular and available in many countries, but they have a number of problems: because they are mostly administered by intradermal or intramuscular injection they can induce only a few antibody responses and therefore cannot protect against the viral infection itself in the upper respiratory tract; it currently takes over 6 months to manufacture them using embryonated chicken eggs; and vaccines derived from H5N1 viruses are not able to be manufactured in embryonated chicken eggs because of their high pathogenicity [6]. In order to address these problems, live attenuated influenza vaccines (LAIVs) have been developed and are currently in use. For example, FluMist, which is one of the LAIVs licensed by the Food and Drug Administration (FDA) in the United States of America (USA), has some advantages over inactivated influenza virus vaccines as they are manufactured from cold-adapted, temperature sensitive, and attenuated IAVs. Therefore, LAIVs are seen as ideal vaccines for prophylaxis of influenza. In order to develop novel LAIVs, it is crucial to understand the molecular basis of the pathogenesis of the IAV.

**Fig 1:**
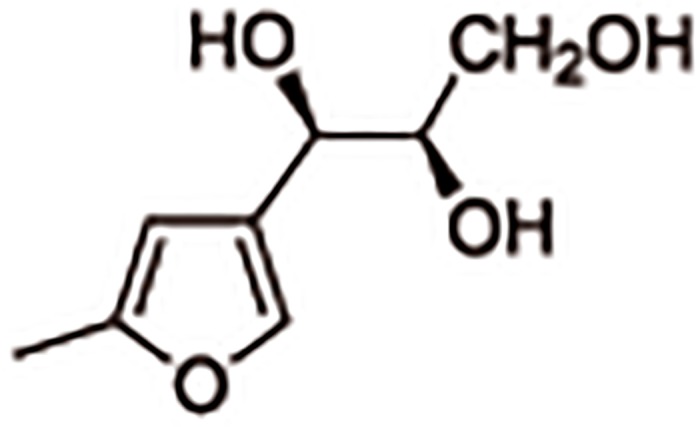
Structure of (1*R*,2*R*)-1-(5’-methylfur-3’-yl)propane-1,2,3-triol (MFPT).

Previously, we have reported that a stable furan derivative, (1*R*,2*R*)-1-(5’-methylfur-3’-yl)propane-1,2,3-triol (MFPT) which has been isolated from *Streptomyces* sp. strain FV60, showed anti-herpes simplex virus type-1 (HSV-1) activity *in vitro*, and that HSV-1 could be resistant to MFPT and result in the abnormal plaque morphology when the viruses were passaged in the presence of MFPT [7]. The structure of MFPT is illustrated in Fig. 1. It is noteworthy that the pathogenicity of the MFPT-resistant (MFPT^r^) HSV-1 was attenuated in the mouse vaginal infection model (unpublished data). These findings prompted us to investigate whether this phenomenon of HSV attenuation could apply to the IAV. From this point of view, we generated MFPT^r^ IAVs by successive passages in the presence of MFPT, investigated their pathogenicity in a mouse model and present in this study the results concerning the mechanisms of lowering the pathogenicity of MFPT^r^ IAVs.

## Materials and Methods

### Ethics statement

All animal experiments were carried out in accordance with the animal experimentation guidelines of the University of Toyama and were approved by the Institute of Animal Care and Use Committee of the University of Toyama (permission number: A2012PHA-18). All infections and sample collections were performed under sodium pentobarbital anesthesia (50 mg/kg), and human endpoints were used during the study, that is, animals with >20% rapid weight loss within 2 days were sacrificed using sodium pentobarbital treatment (150 mg/kg). All efforts were made to minimize animal suffering.

### Cells, viruses and chemicals

Madin-Darby canine kidney (MDCK) cells were grown in Eagle's minimal essential medium (MEM) (Nissui Pharmaceutical Co. Ltd., Tokyo, Japan) supplemented with 5% fetal bovine serum and antibiotics. AX-4 cells [8,9] were cultured in the presence of 7.5 μg/ml of puromycin in MEM supplemented with 5% fetal bovine serum for the selection of plasmid containing the ST6Gal-I gene. An influenza A virus (A/NWS/33, H1N1 subtype) was propagated on MDCK cells and the supernatants were titrated by a plaque assay on MDCK cells and stored at -80°C until use. MFPT was isolated from a *Streptomyces* sp. strain FV60 as previously described [7].

### Generation of MFPT-resistant viruses

MFPT^r^ viruses were generated by 10 passages in the presence of MFPT as follows: MDCK cells in 48 well plates were infected with the NWS strain of IAV at 0.1 plaque-forming unit (PFU) per cell, and incubated at 37°C in the presence of 2 mM of MFPT. After 20 to 24 hours of infection when the full appearance of the cytopathic effect was confirmed, the culture medium was harvested and diluted 10 times with the medium to be subjected to the infection of MDCK cells. After 10 passages, the progeny viruses were plaque-purified on MDCK cell monolayers to obtain the virus clones (MFPT^r^ viruses).

### Growth kinetics of MFPT^r^ viruses *in vitro*

MDCK cells in 48 well plates were infected for 1 hour at room temperature with MFPT^r^ viruses or NWS strain of IAV as wild-type (WT) virus at 1 PFU/cell. The cells were incubated for 30 minutes at 37°C for the viruses to penetrate completely into the cells followed by washing once with phosphate buffered saline (PBS). After treatment with 40 mM citrate buffer (pH 3) for 1 minute in order to inactivate extracellular viruses, the cells were washed with MEM once and incubated at 37°C. The media were collected in each prescribed time, and their virus yields were determined by a plaque assay in triplicate.

### Examination of pathogenicity of MFPT^r^ viruses *in vivo*

Female BALB/c mice 5 to 6 weeks old purchased from the Japan SLC (Shizuoka, Japan) were intranasally infected with MFPT^r^ viruses or the WT virus at 1 × 10^5^ PFU/50 μl/mouse (n = 10) on day 0. Half of the mice were sacrificed at 3 days post-infection, and lung samples and bronchoalveolar lavage fluids (BALFs) were collected. Lung samples were sonicated for 10 seconds after adding 1 μl of PBS per 1 mg of lung tissue, and centrifuged at 5,000 rpm for 10 minutes to separate the supernatants that were stored at -80°C. BALFs were collected by 4 washes with 0.8 ml of ice-cold PBS via a tracheal cannula and centrifuged at 1,500 rpm for 10 minutes to obtain the supernatants that were stored at -80°C. Virus yields of these samples were determined by a plaque assay. Body weight change and survivors of the remaining mice were recorded up to 14 days post-infection.

### Effect of infection (vaccination) with MFPT^r^ viruses on lethal challenge

At 32 days after virus inoculation as described above, surviving mice were intranasally re-challenged with a lethal dose of the WT virus (1 × 10^6^ PFU/50 μl/mouse). As an unvaccinated control group, 3 new mice were similarly infected with the virus. The body weights of the survivors were recorded up to 14 days after re-challenge with the virus. Before re-challenge (at 3 weeks post-infection), blood samples were taken from the tail vein of each surviving mouse, and neutralizing anti-influenza virus antibody titers of the sera were determined using a 50% plaque reduction assay. That is, the approximately 200 PFUs of the WT virus were mixed with sera at 20 to 75,000 fold dilutions with PBS, and incubated at 37°C for 1 hour. Each dilution was added onto MDCK cell monolayers in 35 mm dishes. Plaque numbers in each dish were counted after 2 days to measure the remaining virus infectivity. The neutralizing antibody titer was considered as the highest dilution of the serum that reduced the plaque numbers by 50%, as compared with the control that contained PBS instead of serum.

### Analysis of genome sequences of MFPT^r^ viruses by direct sequencing

MDCK cells in 35 mm dishes were infected with MFPT^r^ viruses or the WT virus and incubated overnight. The viral ribonucleic acid (vRNA) was harvested from the culture medium using QIAamp Viral RNA Mini Kit (QIAGEN K.K., Tokyo, Japan), and reverse transcription-polymerase chain reaction (RT-PCR) was performed to synthesize complementary deoxyribonucleic acid (cDNA) using SuperScript III First-Strand Synthesis System for RT-PCR (Life Technologies, CA, USA) with Uni 12 primer [10]. The cDNA was amplified by polymerase chain reaction (PCR) using segment specific primers as previously reported [11]. PCR products after purification using ExoSAP-IT (GE Healthcare UK Ltd., England) were sequenced using BigDye terminator v3.1 Cycle Sequencing Kit and ABI PRISM 3100 and 3130*xl* Genetic Analyzer (Life Technologies). The sequences of cRNA sequences were deposited in DDBJ (accession numbers: pb2, AB981587; ns1, AB981588).

### Western blotting

MDCK cells in 35 mm dishes were infected with MFPT^r^ virus or the WT virus at 1 PFU/cell or mock infected with PBS. At 0, 5, 10 and 15 hours post-infection, the infected cells were washed 3 times with PBS and stored at -80°C. Using cell lysis buffer (0.05 M Tris-HCl pH 7.0, 0.15 M NaCl, 1% SDS, 1% Triton X-100), the cell extracts were prepared and subjected to 10% SDS-PAGE. The resulting gels were blotted on PVDF membranes and soaked with 2% BSA in TBS for blocking treatment. Blotted proteins were immuno-reacted with rabbit anti-Akt antibody (Cell Signaling Technology, MA, USA), anti-phospho-Akt (Ser473) (D9E) XP Rabbit mAb (Cell Signaling Technology), anti-cleaved caspase 3 (Cell Signaling Technology), anti-PARP-1 (Santa Cruz Biotechnology, TX, USA), anti-Actin(c-2) (Santa Cruz Biotechnology), anti-Influenza A m1 (Santa Cruz Biotechnology), or anti-Influenza A ns1 (Santa Cruz Biotechnology) antibodies. Horseradish peroxidase (HRP)-conjugated goat anti-rabbit IgG or goat anti-mouse IgG antibodies (Santa Cruz Biotechnology) were used as secondary antibodies. Bands of these proteins were detected using TMB Stabilized Substrate for HRP (Promega KK, Tokyo, Japan). Semi-quantifying the expression levels of these proteins were performed using an image analyzing software, ImageJ [12] and that of actin for data normalization.

### Immunofluorescence analysis

MDCK cells (7.6 × 10^5^ cells) in glass base dishes (IWAKI, Asahi Glass Co. Ltd., Chiba, Japan) were infected with MFPT^r^ virus or the WT virus at 1 PFU/cell or mock infected with PBS. At 0, 5, 10, and 15 hours post-infection, the infected cells were fixed using 4% paraformaldehyde phosphate buffer solution, and permeabilized with 0.2% Triton X-100. After blocking treatment with 1% BSA in PBS, the cells were immune-reacted with FITC conjugated-mouse anti-influenza A virus nucleoprotein (431) antibody (Abcam PLC, Cambridge, UK), and nuclei were counterstained by 4’,6-diamidino-2-phenylindole dihydrochloride (DAPI) (Nacalai Tesque, Kyoto, Japan). Stained cells were visualized using fluorescence microscopy, BZ-8000 (Keyence Corp., Osaka, Japan), and obtained images were analyzed by using ImageJ [12].

### Quantitative real-time PCR (qPCR) of interferon-β (IFN-β) mRNA

MDCK cells in 6 well plates were infected with MFPT^r^ viruses or the WT virus at 1 PFU/cell or mock infected with PBS in triplicate. At 10 and 15 hours post-infection, the infected cells were collected and stored at -80°C. Total ribonucleic acid (RNA) from the cells was isolated using RNeasy Mini Kit (QIAGEN K.K.) according to the instructions of the manufacturer. One microgram of isolated RNA was reverse-transcribed to cDNA using SuperScript III First-Strand Synthesis System with Oligo(dT)-primer (Life Technologies). qPCR was performed on Mx3005P (Agilent Technologies, CA, USA) using GoTaq qPCR Master Mix (Promega KK). One microliter of cDNA solution was used in a final reaction volume of 20 μl. Reaction setup and thermal cycling parameters were taken under the manufacturer's instruction with some modifications. Gene expression levels of interferon-β (IFN-β) were calculated by efficiently corrected ΔΔCt method, using 18s rRNA for data normalization and uninfected cells (mock) as the calibrator [13]. The primers were as follows: IFN-β_FOR CCAGTTCCAGAAGGAGGACA; and IFN-β_REV TGTCCCAGGTGAAGTTTTCC, and primers for 18s rRNA were taken from RTPrimerDB (Primer ID 3879) and validated on MDCK cells [14,15].

### Statistical analysis

Data except for animal studies were analyzed for statistical significance by Student’s *t* test or Welch test according to variance equivalency of the each data. In the animal studies, body weights and survival rates of mice were compared by Mann-Whitney U test and log-rank test, respectively.

## Results and Discussion

### Generation and growth kinetics of MFPT^r^ viruses

At first, we generated MFPT^r^ viruses by passing IAVs in the presence of 2 mM MFPT in MDCK cells. After 10 passages, the supernatants of infected cells were plaque-purified on MDCK cell monolayers, and 20 virus clones were obtained as MFPT^r^ viruses. Among them, 10 virus clones, named clone 1 to clone 10, were selected at random and propagated on MDCK cells to use for the subsequent studies.

**Fig 2:**
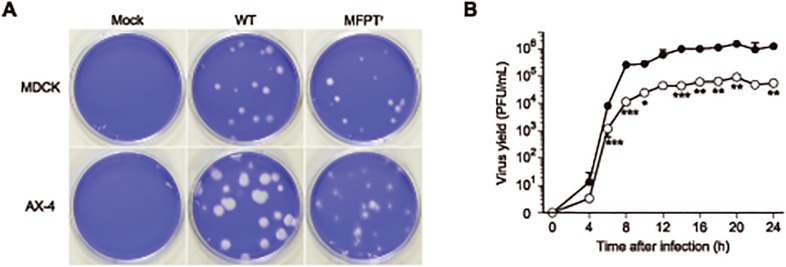
MFPT^r^ virus changes plaque phenotype and reduces viral growth *in vitro*. (A) Plaque phenotypes of the WT virus and MFPT^r^ virus (clone 1) were characterized in MDCK and AX-4 cells. Each cell line was infected with the WT or MFPT^r^ virus at 1 × 10^-5^ PFU/cell or mock infected with PBS. After infection, the cell monolayers were overlaid with the media containing 0.8% agar and incubated at 37°C. At 72 hours post-infection, the cells were fixed and stained by crystal violet. (B) Growth kinetics of the WT virus (closed circle) and MFPT^r^ virus (clone 1, open circle) were measured in MDCK cells infected at 1 PFU/cell. Virus titers were determined by a plaque-reduction assay at the indicated time points. Data were expressed as the mean ± SD of triplicate assays. *p < 0.05, **p <0.01, ***p <0.001.

Fig. 2A shows plaque phenotypes of MFPT^r^ virus (clone 1). The plaque size of MFPT^r^ virus was only slightly reduced compared to that of the WT virus in MDCK cells. The MFPT^r^ virus grown on AX-4 cells, which were introduced ST6Gal-I gene-harboring plasmid, formed significantly smaller plaques than those of the WT virus. ST6Gal-I gene encodes a human β-galactoside α2,6-sialyltransferase I, and AX-4 cells increased the cell surface receptor of IAVs [8]. Therefore, this cell line shows enhanced sensitivity of IAV infection compared to the parent MDCK cells. From these results, the replication efficiency of MFPT^r^ virus was significantly lower than that of the parent virus.

In order to evaluate the growth kinetics of MFPT^r^ viruses, MDCK cells were infected with MFPT^r^ viruses or the WT virus at 1 PFU/cell, and the virus yields in the medium were determined at the times from 0 to 24 hours post-infection. Fig. 2B shows the growth curves of the WT virus and MFPT^r^ virus (clone1) where the yields of progeny viruses of MFPT^r^ virus were decreased compared to that of the WT virus at all the times tested. At 24 hours post-infection, the yield of the MFPT^r^ virus (5.56 × 10^4^ PFU/ml) was approximately 20-fold less than that of the WT virus (1.24 × 10^6^ PFU/ml) with statistical significance (*p* < 0.01). In addition, the growth kinetics of the other MFPT^r^ virus clones showed no significant differences to that of clone 1 (data not shown).

### MFPT^r^ viruses had lower pathogenicity than WT virus

**Fig 3:**
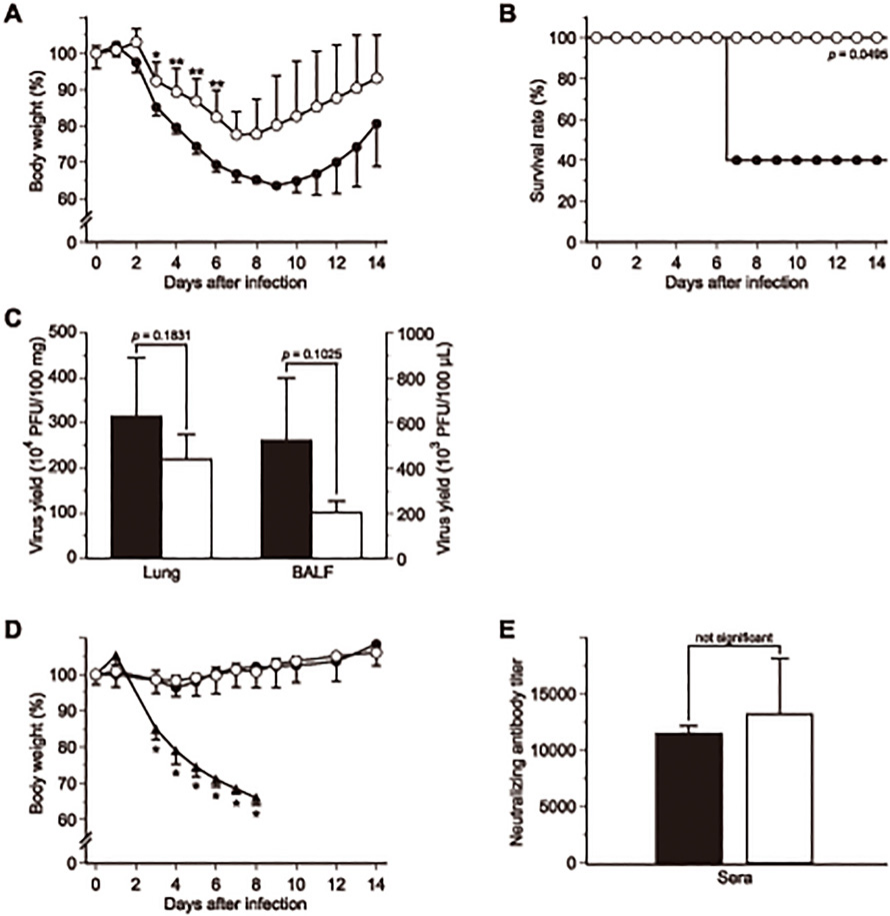
MFPT^r^ virus might be a candidate for live attenuated influenza vaccine. (A) Body weight changes of mice infected with the WT virus (closed circle) or MFPT^r^ virus (clone 1, open circle). BALB/c mice were infected intranasally with the WT or MFPT^r^ virus at 1 × 10^5^ PFU. Data were expressed as the mean ± SD (n = 5). *p < 0.05, **p <0.01, ***p <0.001. (B) Survival rates of mice infected with the WT virus (closed circle) or MFPT^r^ virus (clone 1, open circle). (C) Virus titers in the lungs and BALFs of the WT virus (closed bar) and MFPT^r^ virus (clone 1, open bar) determined at 3 days post-infection. Data were expressed as the mean ± SD. (D) Body weight changes of mice re-challenged with the WT virus in the lethal condition. The WT virus (closed circle, n = 2) or MFPT^r^ virus (clone 1, open circle, n = 5)-inoculated mice were infected intranasally with the WT virus at 1 × 10^6^ PFU. In addition, non-vaccinated 3 mice (closed triangle) were also treated under the same condition. *p < 0.05 (MFPT^r^ virus-inoculated group *versus* non-vaccinated group). (E) Neutralizing antibody titer against the WT virus in sera from the WT virus (closed bar) or MFPT^r^ virus (clone 1, open bar)-inoculated mice at 3 weeks post-infection. Data were expressed as the mean ± SD.

In order to compare the virulence of MFPT^r^ viruses with that of the WT virus, BALB/c mice were intranasally infected with 1 × 10^5^ PFU of MFPT^r^ viruses or the WT virus, and monitored for signs of body weight loss and survival rate. On the one hand, as shown in Fig. 3A, body weights of mice infected with the MFPT^r^ virus (clone 1) decreased by approximately 20% at 8 days post-infection from that on day 0. The weight loss profiles of mice infected with the other MFPT^r^ virus clones were similar to this clone (data not shown). On the other hand, the body weights of WT virus-inoculated mice decreased by approximately 36% at 9 days post-infection. As a result, the mice infected with MFPT^r^ viruses exhibited less morbidity than the mice infected with the WT virus. Notably, 60% of WT virus-inoculated mice reached experimental end point at 7 days post-infection, whereas all the mice of MFPT^r^ virus-inoculated group survived during the observation period and thereafter (Fig. 3B). In order to assess viral replication *in vivo*, lungs and BALFs were collected at 3 days post-infection. Virus yields in the lung and BALF samples obtained from the MFPT^r^ virus-inoculated group tended to be lower than those of WT virus-inoculated group (Fig. 3C). The survival rates and virus yields of the other MFPT^r^ virus-inoculated groups showed a similar tendency to those observed in the MFPT^r^ virus (clone 1)-inoculated group (data not shown). Similar to *in vitro* growth kinetics of the viruses, MFPT^r^ viruses also possessed poor growth efficiency in mice. These results demonstrated that the pathogenic potency of IAVs could obviously be attenuated through treatment with MFPT.

### MFPT^r^ viruses might be an LAIV candidate

As described above, MFPT^r^ viruses had attenuated their pathogenicity in mice. Thus, we expected that the viruses could possess potentiality as LAIV. In order to determine whether prior infection with MFPT^r^ viruses might be sufficient to confer protection against a lethal challenge with IAVs, the mice seroconverted with MFPT^r^ viruses were infected with a lethal dose (1 × 10^6^ PFU) of the WT virus. In this experiment, as non-vaccinated control mice, 3 new ones which had not been infected with IAVs were similarly infected with the WT virus. As shown in Fig. 3D, all the mice previously inoculated with MFPT^r^ virus (clone 1) survived as expected, and no significant changes in body weight were observed in the group. A similar result was also observed in the mice that had survived WT virus infection. In contrast to these groups, the average body weight of non-vaccinated control mice decreased to approximately 66% and they succumbed to virus challenge by day 8 post-infection. When neutralizing antibody titers in the sera of mice at 3 weeks post-infection were determined, the titers of the MFPT^r^ virus (clone 1)-infected group were increased to the levels comparable to those of WT virus-infected group (Fig. 3E). The surviving mice which had be infected with the other MFPT^r^ virus clones also survived after re-challenging and showed high neutralizing antibody titers comparable to those of the WT virus-infected group (data not shown). These results indicated that the MFPT^r^ viruses could induce anti-IAV responses in the animals to cause sufficient protection against the lethal virus challenge. *In vivo* experiment data revealed that the MFPT^r^ viruses had potentiality for the use as LAIV.

### The MFPT^r^ virus carried a single mutation in NS1 protein

In order to validate the genotype of MFPT^r^ viruses, we sequenced their viral genomes (ORFs) by a direct sequencing method [10,11]. According to the sequencing of mutant virus genome (clone 1), 2 mutations, C490U in *ns* gene (P164S in NS1) and G2044U in *pb2* (G682C), were detected by comparing them to that of the parent virus (S1 Fig. and S2 Fig.). It was noteworthy that the former mutation was detected in all MFPT^r^ virus clones, whereas the later was not detected in the other clones (data not shown). Therefore, it was suggested that NS1 might be a main target protein of MFPT and the P164S mutation might be speculated to contribute to the attenuated pathogenicity of MFPT^r^ viruses. In addition, no back mutation (S164P in NS1) was detected after an additional 10 passages in the absence of MFPT (data not shown).

### Function of the MFPT^r^ virus NS1 protein was changed by introducing a mutation

In order to confirm our hypothesis as described above, we investigated using both *in vivo* and *in vitro* systems to determine whether the function(s) of NS1^P164S^ might be changed, and if so, how this mutation could contribute to the attenuation of the pathogenicity of the viruses. In the host cells, NS1 protein has been known to be highly expressed after virus infection and to interact with many host proteins to support viral propagation [16]. As well, it has been revealed that NS1 protein interacts with phosphatidylinositol-3 kinase (PI3K) and solely activates the PI3K/Akt signaling pathway during infection [17]. Moreover, several researchers have also pointed out that the Pro^164^ residue in NS1 is one of the important amino acid residues for the interaction between NS1 and PI3K [18–21]. Therefore, we examined the effects of NS1^P164S^ on the activation of the PI3K/Akt signaling pathway.

**Fig 4:**
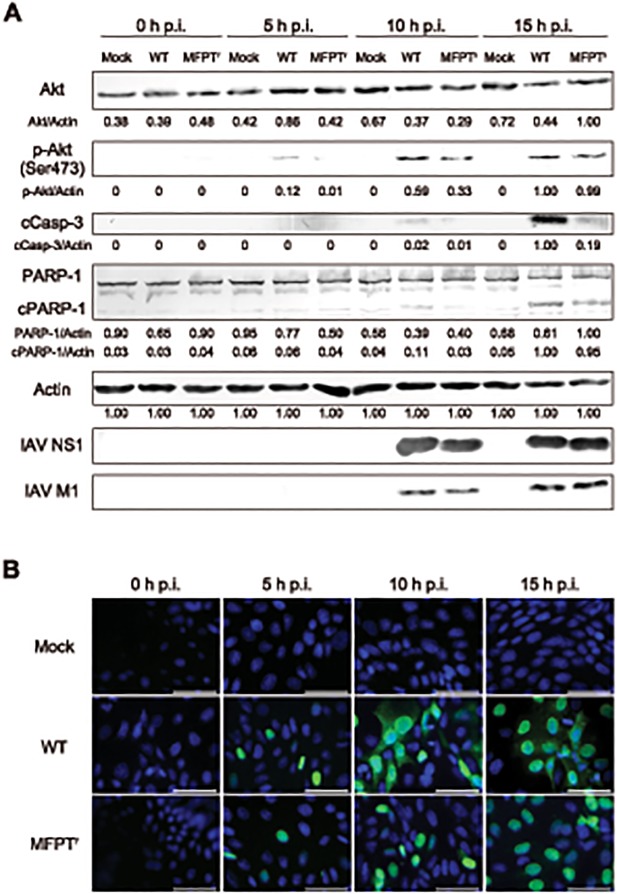
Differences of cellular events between the WT virus and MFPT^r^ virus. (A) MDCK cells were infected with the WT virus or MFPT^r^ virus (clone 1) at 1 PFU/cell or mock infected with PBS. After incubation for the indicated periods, Akt, p-Akt, cleaved caspase-3 (cCasp-3), PARP-1, and cleaved PARP-1 (cPARP-1) were detected by Western blotting. Expression levels of NS1 and M1 were also checked. Semi-quantified expression levels normalized against value for actin were indicated. (B) MDCK cells were infected with the WT virus or MFPT^r^ virus (clone 1) at 1 PFU/cell or mock infected with PBS. After incubation for the indicated periods, IAV NP in the cells was stained with FITC-labeled anti-NP antibody and detected by using immunofluorescence microscopy. Nuclei of cells were counterstained by DAPI. Scale bar = 50 μm. p.i.; post-infection.

As shown in Fig. 4A, Akt was phosphorylated by infection with the WT virus at 5 hours post-infection, and the expression level of phospho-Akt (p-Akt) in MDCK cells infected with MFPT^r^ virus (clone 1) was infallibly lower than that of the WT virus at each time point. Therefore, it was confirmed that the activation of the PI3K/Akt signaling pathway was suppressed by the MFPT^r^ virus infection when compared with that induced by the WT virus. In addition, it was an important finding that there were no significant differences of the expression levels of NS1 and M1 between WT and MFPT^r^ virus-infected cells. This finding revealed that viral protein synthesis such as NS1 and M1 was not affected by the introduced mutation by MFPT and the lowered phosphorylation level of Akt by MFPT^r^ virus infection. This also suggested that the polymerase activity of MFPT^r^ virus might not be affected by another mutation, G682C in PB2. It was reported that Gly^682^ in PB2 is located in a region which binds to PB1 and viral nucleoprotein (NP) to form viral ribonucleoproteins (vRNPs) [22], but the importance of the residue for the function of PB2 is not elucidated at present. Although we cannot completely deny the influence on attenuation of MFPT^r^ virus to depend on G682C in PB2, it cannot be certain that the degree of influence could be small based on the present result.

The PI3K/Akt signaling pathway modulates various cell events such as cell survival, antiviral response and so on [17]. In order to elucidate the differences of NS1 function in WT and MFPT^r^ virus (clone 1), expression levels of cleaved caspase-3 (cCasp-3) and cleaved PARP-1 (cPARP-1) were also analyzed (Fig. 4A). From 10 hours post-infection, expression levels of cCasp-3 and cPARP-1 were increased in a time-dependent manner in WT virus-infected cells. However, the expression levels of these apoptotic markers in MFPT^r^ virus-infected cells were lower than those of WT virus-infected cells. Therefore, it was suggested that the introduced mutation (P164S) in NS1 protein could suppress the progression of apoptosis induced by IAV replication.

So far, several reports revealed that the progression of apoptosis at the late stage in viral replication could support the export of vRNPs from nucleus to cytoplasm and increase virus titer [23–25]. Export of vRNPs occurred by apoptosis could be required for efficient production of progeny viruses, although this has been also mediated by the active cellular export machinery involving activity of the viral nuclear export proteins (NEP/NS2) [2]. Therefore, we attempted to investigate the vRNPs localization in WT virus and MFPT^r^ virus-infected cells by detecting viral NP using the immunofluorescence method. On the one hand, in WT virus-infected cells, NP was solely localized in the nuclei at 5 hours post-infection, and thereafter, NP could be detected in both the nuclei and cytoplasm at 10 and 15 hours post-infection. On the other hand, cytoplasmic localization of NP was hardly detected in MFPT^r^ virus (clone 1)-infected cells at each time point (Fig. 4B). Since it has been shown that NP is mostly forming vRNP complexes in IAV-infected cells [26], our observation suggested that nuclear export of vRNPs could be suppressed in MFPT^r^ virus-infected cells as compared with those of WT virus-infected cells. That is, the suppressed nuclear export of vRNPs might lead to inefficient production of progeny viruses in MFPT^r^ virus-infected cells.

**Fig 5:**
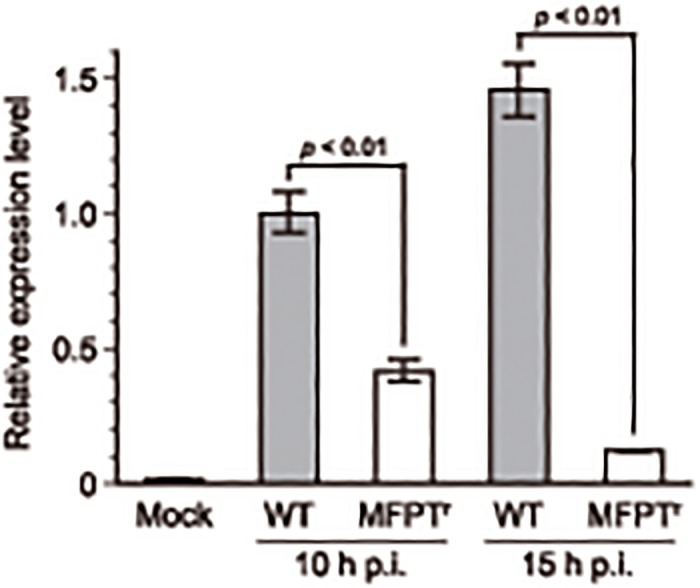
Relative expression level of IFN-β mRNA. MDCK cells were infected with the WT virus or MFPT^r^ virus (clone 1) at 1 PFU/cell or mock infected with PBS in triplicate. At 10 and 15 hours post-infection, the infected cells were collected and the total RNA was isolated followed by RT-PCR to synthesis cDNA. Gene expression levels of IFN-β mRNA were quantified by qPCR. Data were expressed as mean ± SEM. p.i.; post-infection.

The PI3K/Akt signaling pathway also modulates antiviral responses including the induction of IFN-β production. Thus, we examined whether MFPT^r^ virus infection could antagonize the IFN-β induction in the virus-infected cells or not. As shown in Fig. 5, the IFN-β gene expression was clearly induced in WT virus-infected cells at 10 and 15 hours post-infection, whereas it was hardly detected in mock-infected cells. In contrast to the WT virus, IFN-β mRNA expression levels were significantly suppressed in MFPT^r^ virus (clone 1)-infected cells at both time points. The suppression of the mRNA expression levels was also detected in the cells infected with the other MFPT^r^ virus clones (data not shown). It was noteworthy that the expression level of the mRNA at 15 hours post-infection was decreased when compared with that at 10 hours post-infection in MFPT^r^ virus-infected cells. Onomoto *et al*. reported that protein kinase R (PKR) and retinoic acid inducible gene I (RIG- I), which recognize vRNA in cytoplasm and activate antiviral responses, might also recognize vRNA in vRNPs [26]. Other research groups have also reported that RIG- I is an essential component to induce IFN-β production in IAV-infected cells [27–29]. Therefore, we expected that because of suppression of vRNPs levels in cytoplasm in MFPT^r^ virus-infected cells (Fig. 4B), recognition of vRNA by RIG- I in cytoplasm would be decreased. It follows then that RIG- I induced-antiviral responses including IFN-β mRNA expression might be decreased. In addition, because type I interferons including IFN-β induced apoptotic response in IAV-infected cells, the low expression levels of IFN-β mRNA in MFPT^r^ virus-infected cells were consistent with the results of Western blotting (Fig. 4A).

It is important for IAV replication to balance anti-apoptotic and pro-apoptotic states in infected cells since both premature apoptosis at the early stage and suppression of apoptosis at the late stage could reduce virus titer. Shin *et al*. and other researchers reported that IAV which had mutated NS1, NS1^P162A, P164A, P167A^, showed suppression of activation of the PI3K/Akt signaling pathway, low virus titer and premature apoptosis in infected cells [20,21,30]. Both the triple mutated NS1 and our MFPT-induced NS1^P164S^ showed suppression of activation of the PI3K/Akt signaling pathway. However, they had different effects against the progression of apoptosis in the infected cells. We anticipated that these contradictory results might arise from differences in the virus strain (PR8 *versus* NWS), cell type (A549 *versus* MDCK) and so on. Structural studies indicated that Pro^164^ residue in NS1 was shown to lie at an extreme edge of the interface between the NS1 effector domain and the inter-SH2 domain of p85β regulatory subunit in PI3K, effectively closing off the complex [18]. Therefore, the present novel mutation, NS1^P164S^, might be one of a number of key residues to control NS1 function concerning induction of apoptosis.

### The action of MFPT against IAV

Although the attenuated mechanisms of MFPT^r^ virus were investigated, the action of MFPT against IAVs was not confirmed. Thus we attempted to elucidate the action to ensure that NS1 is a main target protein of MFPT. As shown in S3 Fig., the expression level of p-Akt in MDCK cells infected with the WT virus (1 PFU/cell) was decreased at 10 and 15 hours post-infection when MFPT (2 mM) was added to the medium immediately after virus infection. In addition, the expression levels of apoptosis marker proteins such as cPARP-1 and cCasp-3 were also decreased whereas there were no significant changes on those of NS1 and M1 by the treatment with MFPT. These results demonstrated that MFPT could inhibit the activation of the PI3K/Akt signaling pathway that supports viral propagation, and could hardly affect the synthesis of such viral proteins. Also, this suggested that MFPT might inhibit the interaction between PI3K and NS1, because NS1 interacts with PI3K, and solely activates the PI3K/Akt signaling pathway during infection [17]. Therefore, it was thought that IAVs would mutate the NS1 protein to adapt its replication in the presence of MFPT, resulting in a mutation of Pro^164^ in NS1 which is one of the important amino acid residues for the interaction between NS1 and PI3K.

## Conclusion

In this paper we investigated the mechanism of lowering the pathogenicity of the IAV by treating it with MFPT, a stable furan derivative. Although the antiviral activity of MFPT against IAVs was not a potent one (the 50% inhibitory concentration for virus replication = 2.8 mM), MFPT^r^ viruses were attenuated both *in vitro* and *in vivo* as expected, and had a common novel mutation, P164S in NS1. The study showed that, unfortunately, the pathogenicity of the attenuated virus might be a little strong to use itself as a viral strain for LAIV (Fig. 3A and 3C). However, there could be potentiality to increase the safety of the LAIV by combining this mutation with other mutations associated with attenuation of IAVs. As well, it is important to elucidate the mechanisms associated with the virulence/pathogenicity for more deeply understanding the nature of IAVs. For these reasons, we contend that this study is an important step in the continuing development of attenuated vaccines for the prophylactic treatment of influenza.

## Supporting Information

S1 Fig Nucleotide sequence of ns (A) and amino acid sequence of ns1 (B). (PDF)

S2 Fig Nucleotide sequence (A) and amino acid sequence (B) of pb2. (PDF)

S3 Fig Inhibition of the activation of the PI3K/Akt signaling pathway by MFPT treatment. (PDF)
