# Supplementary material for: Characterization of a Novel Mutation in NS1 Protein of Influenza A Virus Induced by a Chemical Substance for the Attenuation of Pathogenicity
Source: PLoS One. 2015 Mar 20;10(3):e0121205. doi: 10.1371/journal.pone.0121205 (PMC4368802; doi:10.1371/journal.pone.0121205)
Supplement: S1 Fig — (PDF) [file pone.0121205.s001.pdf]

A

WT virus AUGGAUCCAAACACUGUGUCAAGCUUUCAGGUAGAUUGCUUUCUUUGGCAUGUCCGCAAAAGAGUUGCAGACCAAGAACUAGGUGAUUCCCCAUUCCUUG 100
MFPT^{r} virus 100

WT virus AUCGGCUUCGCCGAGAUCAGAAGUCCCUAAGAGGAAGAGGCAGCACUCUCGGUCUGGACAUCGAAACAGCCACCCGUGCUGGAAAGCAAAUAGUGGAGCG 200
MFPT^{r} virus 200

WT virus GAUUCUGAAGGAAGAAUCCGAUGAGGCACUUAAAAUGACCAUGGCCUCUGUACCUGCGUCGCGCUACCUAACUGACAUGACUCUUGAGGAAAUGUCAAGG 300
MFPT^{r} virus 300

WT virus CACUGGUUCAUGCUCAUGCCCAAGCAGAAAGUGGCUGGCCCUCUUUGUAUCAGAAUGGACCAGGCGAUCAUGGAUAAGAACAUCAUACUGAAAGCGAACU 400
MFPT^{r} virus 400

WT virus UCAGUGUGAUUUUUGACCGGCUGGAGACUCUAAUAUUACUAAGGGCCUUCACCGAAGAGGGAACAAUUGUUGGUGAAAUUUCACCACUGCCUUCUCUUCC 500
MFPT^{r} virus ....U.......... 500

WT virus AGGACAUACUGAUGAGGAUGUCAAAAAUGCAGUUGGGGUCCUCAUCGGAGGACUUGAAUGGAAUAAUAACACAGUUCGAGUCUCUGAAACUAUACAGAGA 600
MFPT^{r} virus 600

WT virus UUCGCUUGGAAAAGCAGUAAUGAGAAUGGGAGACCUCCACUCACUCCAAAACAGAAACGGAAAAUGGCGGGAACAAUUAGGUCAGAAGUUUGAAGAAAUA 700
MFPT^{r} virus 700

WT virus AGAUGGUUGAUUGAAGAAGUGAGACACAGACUGAAGAUAACAGAGAAUAGUUUUGAGCAAAUAACAUUUAUGCAAGCCUUACAACUAUUGCUUGAAGUGG 800
MFPT^{r} virus 800

WT virus AGCAAGAGAUAAGAACUUUCUCGUUUCAGCUUAUUUAA 838
MFPT^{r} virus 838

B

WT virus MDPNTVSSFQVDCFLWHVRKRVADQELGDSPFLDRLRRDQKSLRGRGSTLGLDIETATRAGKQIVERILKEESDEALKMTMASVPASRYLTDMTLEEMSR 100
MFPT^{r} virus 100

WT virus HWFMLMPKQKVAGPLCIRMDQAIMDKNIILKANFSVIFDRLETLILLRAFTEEGTIVGEISPLPSLPGHTDEDVKNAVGVLIGGLEWNNNTVRVSETIQR 200
MFPT^{r} virus ....S.................................... 200

WT virus FAWKSSNENGRPPLTPKQKRKMAGTIRSEV 230
MFPT^{r} virus 230
