# Supplementary material for: Characterization of a Novel Mutation in NS1 Protein of Influenza A Virus Induced by a Chemical Substance for the Attenuation of Pathogenicity
Source: PLoS One. 2015 Mar 20;10(3):e0121205. doi: 10.1371/journal.pone.0121205 (PMC4368802; doi:10.1371/journal.pone.0121205)
Supplement: S2 Fig — (PDF) [file pone.0121205.s002.pdf]

A

WT virus	AUGGAAAGAAUAAAAGAACUAAGGAAUCUAAUGUCGCAGUCUCGCACUCGCGAGAUACUCACAAAAACCACCGUGGACCAUAUGGCCAUAAUCAAGAAGU	100
MFPT^r virus	....	100
WT virus	ACACAUCAGGAAGACAGGAGAAGAACCCAGCACUUAGGAUGAAAUGGAUGAUGGCAAUGAAAUAUCCAAUUACAGCAGACAAGAGGAUAACGGAAAUGAU	200
MFPT^r virus	....	200
WT virus	UCCUGAGAGAAAUGAGCAAGGACAAACUUUAUGGAGUAAAAUGAAUGACGCCGGAUCAGACCGAGUGAUGGUAUCACCUCUGGCUGUGACAUGGUGGAAU	300
MFPT^r virus	....	300
WT virus	AGGAAUGGACCAGUGACAAGUACAGUCCAUUAUCCAAAAAUCUACAAAACUUAUUUUGAAAAAGUCGAAAGGUUAAAACAUGGAACCUUUGGCCCUGUCC	400
MFPT^r virus	....	400
WT virus	AUUUUAGAAACCAAGUCAAAAUACGUCGAAGAGUUGACAUAAAUCCUGGUCAUGCAGAUCUCAGUGCCAAAGAGGCACAGGAUGUAAUCAUGGAAGUUGU	500
MFPT^r virus	....	500
WT virus	UUUCCCCAACGAAGUGGGAGCCAGGAUACUAACAUCGGAAUCGCAACUAACGAUAACCAAAGAGAAGAAAGAAGAACUCCAGGGUUGCAAAAUUUCUCCU	600
MFPT^r virus	....	600
WT virus	CUGAUGGUGGCAUACAUGUUGGAAAGAGAACUGGUCCGGAAAACGAGAUUCCUCCCAGUGGCUGGUGGAACAAGCAGUGUGUACAUUGAAGUGUUGCAUU	700
MFPT^r virus	....	700
WT virus	UGACCCAAGGAACAUGCUGGGAACAGAUGUACACUCCAGGAGGGGAGGUGAGGAAUGAUGAUGUUGAUCAAAGCUUAAUUAUUGCUGCUAGAAACAUAGU	800
MFPT^r virus	....	800
WT virus	AAGAAGAGCCACAGUAUCAGCAGACCCACUAGCAUCUUUAUUGGAGAUGUGCCACAGCACGCAGAUUGGUGGAAUAAGGAUGGUAGACAUCCUUAGGCAG	900
MFPT^r virus	....	900
WT virus	AACCCAACAGAAGAGCAAGCCGUGGAUAUAUGCAAGGCUGCAAUGGGACUGAGAAUUAGCUCAUCCUUCAGUUUUGGUGGAUUCACAUUUAAGAGAACAA	1000
MFPT^r virus	....	1000
WT virus	GCGGAUCAUCAGUCAAGAGAGAGGAAGAGGUGCUUACGGGCAAUCUUCAGACAUUGAAGAUAAGAGUGCAUGAGGGAUAUGAAGAGUUUACAAUGGUUGG	1100
MFPT^r virus	....	1100
WT virus	GAGAAGAGCAACAGCUAUACUCAGAAAAACAACCAGGAGAUUGAUUCAGCUGAUAGUGAGUGGAAGAGACGAACAGUCGAUUGCCGAAGCAAUAAUUGUG	1200
MFPT^r virus	....	1200
WT virus	GCCAUGGUAUUUUCACAAGAGGAUUGUGUGAUAAAAGCAGUUAGAGGUGACCUGAAUUUCGUCAAUAGGGCGAAUCAGCGAUUGAAUCCCAUGCACCAAC	1300
MFPT^r virus	....	1300
WT virus	UUUUGAGACAUUUUCAGAAGGAUGCAAAAGUGCUCUUUCAAAAUUGGGGAACUGAAUCCAUCGACAAUGUGAUGGGAAUGAUUGGGAUAUUGCCCGACAU	1400
MFPT^r virus	....	1400
WT virus	GACUCCAAGCACCGAGAUGUCAAUGAGAGGAGUGAGAGUCAGCAAAAUGGGGGUAGAUGAGUAUUUCAGCGCGGAGAAGGUAGUGGUGAGCAUUGACCGU	1500
MFPT^r virus	....	1500
WT virus	UUUUUGAGAGUUAGGGACCAACGUGGGAAUGUACUACUGUCUCCCGAGGAGGUCAGUGAAACACAGGGGACAGAGAAACUGACAAUAACUUACUCAUCGU	1600
MFPT^r virus	....	1600
WT virus	CAAUGAUGUGGGAGAUUAAUGGUCCUGAAUCAGUGUUGAUCAAUACCUAUCAGUGGAUCAUCAGAAACUGGGAAACUGUUAAAAUUCAGUGGUCCCAGAA	1700
MFPT^r virus	....	1700
WT virus	UCCUACAAUGCUGUACAAUAAAAUGGAAUUUGAGCCAUUUCAGUCUUUAGUUCCAAAGGCCGUUAGAGGCCAAUACAGUGGGUUUGUGAGAACUCUGUUC	1800
MFPT^r virus	....	1800
WT virus	CAACAAAUGAGGGAUGUGCUUGGGACAUUUGAUACUGCUCAGAUAAUAAAACUUCUUCCCUUCGCAGCCGCUCCACCAAAGCAAAGUAAAAUGCAAUUCU	1900
MFPT^r virus	....	1900
WT virus	CCUCAUUGACUGUGAAUGUGAGGGGAUCAGGAAUGAGAAUACUUGUAAGGGGCAAUUCUCCAGUAUUCAACUACAACAAGACCACUAAAAGACUCACAGU	2000
MFPT^r virus	....	2000
WT virus	UCUCGGAAAGGAUGCUGGCACUUUAACUGAAGACCCAGAUGAAGGCACAGCUGGAGUUGAGUCCGCAGUUCUAAGAGGAUUCCUCAUUCUGGGUAAAGAA	2100
MFPT^r virus	....U..	2100
WT virus	GACAGGAGAUAUGGACCAGCAUUAAGCAUAAAUGAACUGAGCAACCUUGCGAAAGGAGAGAAGGCUAAUGUGCUAAUUGGGCAAGGAGACGUGGUGUUGG	2200
MFPT^r virus	....	2200
WT virus	UAAUGAAACGGAAACGGAACUCUAGCAUACUUACUGACAGCCAGACAGCGACCAAAAGAAUUCGGAUGGCCAUCAAUUAG	2280
MFPT^r virus	....	2280

B

WT virus	MERIKELRNLMSQSRTREILTKTTVDHMAIIKKYTSGRQEKNPALRMKWMMAMKYPITADKRITEMIPERNEQGQTLWSKMNDAGSDRVMVSPLAVTWWN	100
MFPT^r virus	....	100
WT virus	RNGPVTSTVHYPKIYKTYFEKVERLKHGTFGPVHFRNQVKIRRRVDINPGHADLSAKEAQDVIMEVVFPNEVGARILTSESQLTITKEKKEELQGCKISP	200
MFPT^r virus	....	200
WT virus	LMVAYMLERELVRKTRFLPVAGGTSSVYIEVLHLTQGTCWEQMYTPGGEVRNDDVDQSLIIAARNIVRRATVSADPLASLLEMCHSTQIGGIRMVDILRQ	300
MFPT^r virus	....	300
WT virus	NPTEEQAVDICKAAMGLRISSSFSFGGFTFKRTSGSSVKREEEVLTGNLQTLKIRVHEGYEEFTMVGRRATAILRKTTRRLIQLIVSGRDEQSIAEAIIV	400
MFPT^r virus	....	400
WT virus	AMVFSQEDCVIKAVRGDLNFVNRANQRLNPMHQLLRHFQKDAKVLFQNWGTESIDNVMGMIGILPDMTPSTEMSMRGVRVSKMGVDEYFSAEKVVVSIDR	500
MFPT^r virus	....	500
WT virus	FLRVRDQRGNVLLSPEEVSETQGTEKLTITYSSSMMWEINGPESVLINTYQWIIRNWETVKIQWSQNPTMLYNKMEFEPFQSLVPKAVRGQYSGFVRTLF	600
MFPT^r virus	....	600
WT virus	QQMRDVLGTFDTAQIIKLLPFAAAPPKQSKMQFSSLTVNVRGSGMRILVRGNSPVFNYNKTTKRLTVLGKDAGTLTEDPDEGTAGVESAVLRGFLILGKE	700
MFPT^r virus	....C..................	700
WT virus	DRRYGPALSINELSNLAKGEKANVLIGQGDVVLVMKRKRNSSILTDSQTATKRIRMAIN	759
MFPT^r virus	....	759
