# Supplementary figures and images for: Characterization of a Novel Mutation in NS1 Protein of Influenza A Virus Induced by a Chemical Substance for the Attenuation of Pathogenicity
Source: PLoS One. 2015 Mar 20;10(3):e0121205. doi: 10.1371/journal.pone.0121205 (PMC4368802; doi:10.1371/journal.pone.0121205)

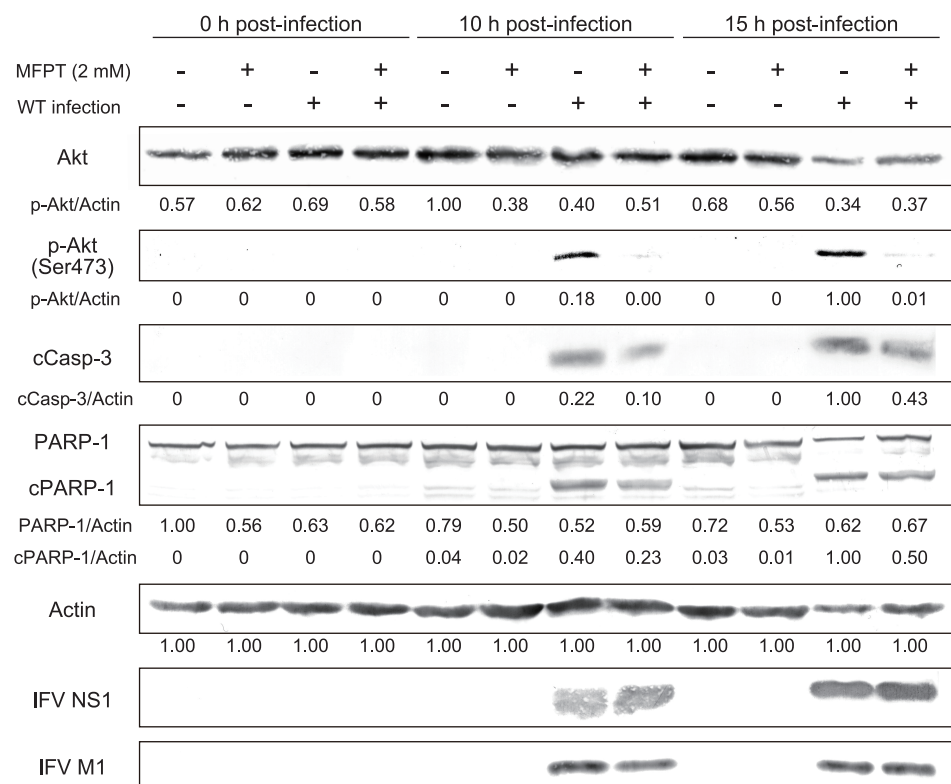

Supplement: S3 Fig — (PDF) [file pone.0121205.s003.pdf]
